# Pediatric subspecialty telemedicine use from the patient and provider perspective

**DOI:** 10.1038/s41390-021-01443-4

**Published:** 2021-03-22

**Authors:** Rajdeep Pooni, Natalie M. Pageler, Christy Sandborg, Tzielan Lee

**Affiliations:** Lucile Packard Children’s Hospital at Stanford, Stanford University School of Medicine, Palo Alto, CA USA

## Abstract

**Background:**

To characterize telemedicine use among pediatric subspecialties with respect to clinical uses of telemedicine, provider experience, and patient perceptions during the COVID-19 pandemic.

**Methods:**

We performed a mixed-methods study of telemedicine visits across pediatric endocrinology, nephrology, orthopedic surgery, and rheumatology at a large children’s hospital. We used deductive analysis to review observational data from 40 video visits. Providers and patients/caregivers were surveyed around areas of satisfaction and communication.

**Results:**

We found adaptations of telemedicine including shared-screen use and provider-guided parent procedures among others. All providers felt that it was safest for their patients to conduct visits by video, and 72.7% reported completing some component of a clinical exam. Patients rated the areas of being respected by the clinical staff/provider and showing care and concern highly, and the mean overall satisfaction was 86.7 ± 19.3%.

**Conclusions:**

Telemedicine has been used to deliver care to pediatric patients during the pandemic, and we found that patients were satisfied with the telemedicine visits during this stressful time and that providers were able to innovate during visits. Telemedicine is a tool that can be successfully adapted to patient and provider needs, but further studies are needed to fully explore its integration in pediatric subspecialty care.

**Impact:**

- This study describes telemedicine use at the height of the COVID-19 pandemic from both a provider and patient perspective, in four different pediatric subspecialties.
- Prior to COVID-19, pediatric telehealth landscape analysis suggested that many pediatric specialty practices had pilot telehealth programs, but there are few published studies evaluating telemedicine performance through the simultaneous patient and provider experience as part of standard care.
- We describe novel uses and adaptations of telemedicine during a time of rapid deployment in pediatric specialty care.

## Introduction

Multiple pediatric subspecialties have struggled with direct patient to provider access. According to the American Academy of Pediatrics, more pediatric subspecialists are working at academic centers located in urban areas, are spending less time face to face with patients, and for some specialties such as endocrinology and neurology, appointment wait times exceed 2 weeks.^1^ In addition, some specialties, such as pediatric rheumatology, do not have practicing providers in every state.^2^ A recent study among families of children and youth with special health care needs, policy experts, and researchers indicated that further research in clinical-model refinement, specifically telemedicine, is of particularly high priority.^3^ As early as 1978, pediatric providers have leveraged the use of telehealth to increase access to tertiary care sites and providers^4^ both as a means to extend outpatient^5,6^ and acute inpatient specialty care.^7,8^ Multiple models of telemedicine care have been described in specialties such as pediatric cardiology,^9,10^ endocrinology,^11,12^ orthopedic surgery,^13^ and palliative care^14^ among others. Studies on the use of telemedicine in pediatric outpatient care in the United States have revealed potential cost-savings to families,^15^ increased health-related quality of life and satisfaction,^16^ and less school and work time missed.^17^ In spite of the steady growth of pediatric telemedicine use in the United States, barriers to increased adoption of telemedicine services had previously included licensing issues, paucity of provider interest, and lack of resources to train providers. For sites that had already incorporated telemedicine practices, reimbursement had remained a challenge and further limited expansion of routine telemedicine practices.^18^ Even for those programs with early telemedicine adoption and policies, telemedicine utilization among patients in pediatric subspecialties has been low.^19^

The COVID-19 pandemic, in many ways, removed these barriers to the implementation of telemedicine resulting in a rapid change in delivery of care across all sectors of health care in response to public health mandates to limit exposures of patients and health care workers to the COVID-19 infection.^20^ Rapid deployment of telemedicine has occurred in academic health systems and hospital networks^21^ leading to incredibly fast implementation across health systems as a substitute for in-person visits. At Stanford Children’s Hospital, a large tertiary care site, the number of pediatric telemedicine visits increased almost 35-fold from over a year ago; each specialty in this study had five or fewer telemedicine visits in April 2019 compared to up to 753 telemedicine visits in April 2020 (N. Pageler and T. Lee, Stanford Children’s Health, 2020). The rapid increase in pediatric subspecialty telemedicine visits has revealed potential benefits and challenges in managing children with chronic disease via telemedicine visits.^22^ In many ways, the COVID-19 pandemic has resulted in a natural experiment in that there are adequate volumes of telemedicine^23^ use across multiple pediatric specialties in order to evaluate telemedicine performance when institutional barriers are removed. We took this opportunity to evaluate the nuances of telemedicine with respect to caregiver–patient–provider communication, patient and provider experience, and clinical approaches to video visits in four different pediatric subspecialties during the rapid deployment of telemedicine at an academic children’s hospital.

## Methods

### Study population

This study is a cross-sectional mixed-methods study of patients, families, and providers participating in telemedicine pediatric subspecialty follow-up visits between March 23, 2020 and May 05, 2020 during the COVID-19 pandemic and was approved by the Stanford University Institutional Review Board. This study was conducted at a large, single academic children’s hospital and satellite clinics and included four subspecialties: pediatric endocrinology, pediatric nephrology, pediatric orthopedic surgery, and pediatric rheumatology. These specialties were selected as they may present unique needs such as postsurgical care, the need for specific types of bloodwork, a specific clinical exam, and varied medication monitoring needs. A convenience sample of ten patients and their caregivers (if patients were <18 years of age) from each specialty were recruited prior to their regularly scheduled video visit appointment with their specialty provider (*n* = 40). Each patient or patient/caregiver set in the study participated in a single telemedicine visit during the course of this study, although specialty providers may have participated in more than one observed telemedicine visit. The telemedicine visits were conducted via one of three platforms based on provider preference: via the Extended Care telehealth platform (Cisco Systems, San Jose, CA) integrated with the EHR (Epic Systems, Verona, WI), via Webex video communications platform (Cisco Systems, San Jose, CA), or via standard Zoom video communications platform (Zoom Video Communications, San Jose, CA). Neither patients/caregivers nor providers had additional training regarding telemedicine visits although they may have received specific instructions on connecting to visits and/or troubleshooting connectivity issues. These workflows varied by department. Exclusion criteria included new patient referrals and consultations, subjects under the age of 5 years, non-English-speaking subjects or non-English-speaking guardians, subjects who canceled or no-showed their scheduled appointments following consent into the study, and subjects who were unable to connect to the video visit platform and declined such visits prior to the consenting process. New referral type and young patient age were specifically excluded as triage practices for these patients varied by specialty.

### Observational data

A total of 40 video visits were observed, ten per specialty. The observer was able to either (1) log in remotely to the visit if the visit used a multiparty telemedicine platform or (2) be in the clinic room with the provider and the patient/family on video. This was dependent on multiparty function capabilities of the platform or last-minute changes to the telemedicine platform. All patients and caregivers were aware that the observer was present but not taking part in the visit. The observer documented visit specifics including (1) physical exam performance and vitals documentation, (2) patient–provider dialogue/communication, (3) ancillary needs including laboratory, social work, nursing, imaging, or other clinical needs, (4) communication regarding the medical assessment and plan, and (5) discussion/communication with other providers present for the visit. These transcripts/observations were reviewed by a second medical reviewer, who employed deductive coding and calculated the results based on a predeveloped checklist of criteria (see Supplementary Material, Fig. 1).

### Survey tools

Following their respective video visit, subjects were sent electronic versions of the Makoul Communication Assessment Tool (Makoul CAT)^24^ and RAND VSQ9^25^ via REDCap. The Makoul CAT measures patient/caregivers’ perceptions of physicians’ communication skills and the VSQ9 is a visit-specific satisfaction survey. In addition, a 13-item provider survey (see Supplementary Material, Fig. 2) was developed for use in this research project via Qualtrics (Qualtrics, Provo, UT) and was distributed to providers following their first study visit. As most providers performed more than one video visit per day, surveys were not patient-encounter-specific. Free response questions included such items as “what exam components, if any, were completed in the visit,” or “what further information would have been needed to make a clinical assessment.” Providers were also queried about their comfort with telemedicine use with such questions as “In the setting of recent public health concerns, was it safer for your patient to conduct these visits by video rather than in person,” which generated binary responses (Yes/No), and percentages were tabulated. Providers gave their overall impression of the quality of the visit using a visual analog scale.

### Data analysis

Analysis for the observational data included simple statistics based on the results of the deductive coding (assigning predefined codes to the qualitative data) and predeveloped criteria checklist (see Supplementary Material, Fig. 1). Analysis for the VSQ9 included overall satisfaction scores by subject evaluated in SAS University Edition (SAS Institute, Cary, NC) to examine differences in scores by subspecialty. Proportions of patients that rated their communication as “excellent” as well as overall scores for the Makoul CAT were also calculated in SAS to detect differences. Provider survey analysis included calculation of the mean and standard deviation.

## Results

### Study participants

A total of 40 participants, ten per specialty, were enrolled in this study. Table 1 shows mean age and sex of participants by specialty. There were a greater number of subjects who identified as female in this study, and although there were no significant imbalances of sex or age between specialties, differences in age may reflect the specialty diseases’ age preference. A range of diagnoses were seen by each subspecialty group during the course of this study and included both acute processes and chronic diagnoses (Table 2).

**Table 1:** Age and sex of study subjects by subspecialty.

Patients by specialty	*N*	Sex	Mean age ± SD (years)
Total	40	31 F, 9 M	13.7 ± 4.0
Endocrinology	10	9 F, 1 M	12.7 ± 4.5
Nephrology	10	7 F, 3 M	14.2 ± 4.1
Orthopedic surgery	10	6 F, 4 M	11.9 ± 3.3
Rheumatology	10	9 F, 1 M	16.1 ± 3.3

*F* female, *M* male.

**Table 2:** Diagnoses seen by specialty.

Endocrinology	Nephrology	Orthopedic Surgery	Rheumatology
Graves disease	C3 glomerulonephritis	Elbow fracture	Mixed connective tissue disease
Premature adrenarche	Chronic kidney disease^a^	Knee pain	Spondylarthritis
Prediabetes	Secondary hypertension	Scoliosis^a^	Arthralgia
Short stature	Nephrotic syndrome	Forearm fractures	Positive ANA
Hypothyroidism^a^	End-stage renal disease^a^	ACL injury	Oligoarticular JIA and uveitis
MEN1		Osteogenesis imperfecta	Systemic lupus erythematosus^a^
		Patellar fracture	Polyarticular JIA^a^
		Supracondylar fracture s/p pinning	
		Postop, hallux valgus correction	

*MEN1* multiple endocrine neoplasia type I, *JIA* juvenile idiopathic arthritis.

^a^More than 1 visit with this primary diagnosis.

### Observations

A total of 40 visits, ten per specialty, were observed during the course of this study. Each visit was coded, and results included the following:

- *Clinic processes*: Using the observation checklist, Table 3 shows a summary of clinic processes observed during the video visits. Of the total visits, 17.5% had technical issues noted, such as patients having difficulty logging in or poor audio quality, but all visits observed in this study were completed. Technical issues may have included 12.5% of visits had clinical staff check-ins completed (e.g., by a medical assistant), and in 30% of visits the caregiver or parent provided vitals. Of the total visits, 67.5% included a focused physical exam, which included provider instruction for examination of various organ systems including skin, respiratory, musculoskeletal, neurologic, and other exam components, and completed by the patient.

**Table 3:** Telemedicine visit observations^a^.

Clinical process	Completed	Incomplete	% Completion
Patient check-in process for visit	5	35	12.5
Focused physical exam by provider	27	13	67.5
Vitals provided by patient	12	28	30.0

^a^This information includes clinical processes related to check-in, exam, and procedures during telemedicine follow-up visit. Results were tabulated using checklist in Figure 1, Appendix.
- *Novel uses of telemedicine*: The observation checklist included descriptive text regarding the use of video visits with respect to technological advancements or provider-led adaptations for telemedicine. These adaptations include clinical activities that are only able to be performed via a combination of telemedicine platform and electronic medical record (EMR) use or clinical adaptations that were completed in response to the patient’s home environment. Of note, there were no specific remote/facilitated exam tools (such as a digital stethoscope) used in these visits. The results are summarized in Table 4 and include novel tasks such as guided cast removal (using simple scissors) and “shared-screen” function use to review growth charts and labs.

**Table 4:** Novel uses of telemedicine^a^.

Technology-assisted adaptations	Provider-led adaptations
Utilization of shared blood pressure logs	Review of medications with prescription bottles in hand
Use of video chat box to clarify feeding prescription information	Multidisciplinary visit
Multidisciplinary^b^ conference prior to patient visit	Demonstration of hygiene care for lines
Review of exit site photos inpatient-provider portal in real-time	Growth pattern reviews with use of clothing/shoe sizes
Provider review of growth curves and labs in real-time via “shared-screen” function	Provider to patient instruction on simple neurologic exam
	HEADSS assessment
	Guided cast removal

^a^Technology-assisted adaptations refer to those items performed in clinical visits that were only able to be performed via a combination of telemedicine platform and EMR use. Provider-led adaptations describe provider-led exam or HPI components completed in part due to technology use and in part due to patient’s home environment.

^b^Multidisciplinary visits refer to visits that comprise more than one provider type. For example, this may include the specialty nutritionist, nurse practitioner, or physician among provider types.
- *Patient- vs parent-led communication*: Of the 40 visits, over a third (37.5%) of the visits involved the patient leading the conversation with the provider.

### Patient communication and satisfaction

The subject response rate for the Makoul CAT was 55 and 50% for the VSQ9. On the Makoul CAT the highest-rated items by the proportion of subjects who rated “excellent” as well as by the overall mean score are shown in Table 5 and included elements such as “treated me with respect,” “showed care and concern,” and “the doctor’s staff treated me with respect.” For the VSQ9, the highest-rated areas of satisfaction included “the personal manner (courtesy, respect, sensitivity, friendliness) of the person you saw” and the “technical skills (thoroughness, carefulness, competence) of the physician/health care professional you saw today.” Lowest-rated items included “getting through to the office by phone” and “how long you waited to get an appointment.”

**Table 5:** Highest-rated items by question on Makoul CAT and VSQ9^a^.

Makoul CAT top rated	VSQ9 top rated
The doctor’s staff treated me with respect	The personal manner (courtesy, respect, sensitivity, friendliness) of the person you saw
Showed care and concern	Technical skills (thoroughness, carefulness, competence) of the physician/health care professional you saw
Treated me with respect	Time spent with the physician/health care professional that you saw

^a^These items reflect the highest-rated line items per questionnaire.

### Provider survey

The provider response rate was 91.6% (*n* = 22) and included providers from the four specialties. Provider reported survey outcomes assessed provider satisfaction and experience with video visits with respect to quality, clinical assessment, and patient safety during the recent public health emergency. One hundred percent of providers included in this survey felt that the video visit was a safer medium to conduct their clinical visit given the recent pandemic. In general, most providers (86.1%) identified that they were able to gather the appropriate information to make a clinical assessment and overall rated the quality of the visit highly (mean 8.1 on a scale of 1–10). In addition, 72.7% of providers reported that they were able to complete elements of the physical exam virtually. Components reported to have been completed included exams predominantly based on inspection (e.g., color, behavior, respirations, appearance of catheter site), although providers also noted specific exam features such as “parts of the neurologic exam” or “joint range of motion.” Several providers also reported measures such as blood pressure and weight. For the free response questions, the response rate was ~50% and not amenable to standard qualitative analysis. Providers had a range of answers regarding their video visit experience: some indicated positive clinical aspects of telemedicine visits (e.g., being able to observe patient in home environment), other responses included technical issues (e.g., “...virtual waiting room was a little unclear,”), and others relayed their overall feelings (both positive and negative) regarding telemedicine use.

## Discussion

This study simultaneously evaluated telemedicine performance with respect to patient–provider communication, provider experience, and necessary clinical adaptations of telemedicine visits across multiple pediatric subspecialties during the COVID-19 pandemic. Amongst our findings, we determined novel uses and nontraditional applications of telemedicine during subspecialty visits that were unique to the experience of providers and patients engaging through a virtual platform as well as a construct of patients being in the home environment. We were also able to demonstrate provider acceptability during this global pandemic and have identified important areas of exploration in regard to patient communication and engagement, which are known to be imperative in patient activation, shared decision-making, and health quality outcomes.^26–28^ Prior to the pandemic, studies had indicated that on some level, telemedicine use in clinical care was acceptable to patients and caregivers,^29–32^ although further work on clinical outcomes and provider acceptability was needed.^33^ Telemedicine adoption can sometimes be difficult, particularly in pediatric subspecialty care due to concern regarding patient safety and potentially provider confidence in being able to make a clinically sound assessment by video visit. The COVID-19 global pandemic, in many ways, reduced traditional implementation barriers to telemedicine such as reimbursement issues^34^ allowing researchers to assess its performance and feasibility in a way that was previously challenging in this vulnerable population. Not surprisingly, this study demonstrated that given the recent public health concerns, all providers felt that it was safer for these patients to be seen via telemedicine as opposed to in-person clinical visits during the pandemic. It also demonstrated that the majority of providers believed that they were able to elicit the necessary information to conduct some level of a focused physical exam and develop an adequate clinical assessment. This was true across all four pediatric specialties. This level of confidence was encouraging given that there are few guidelines developed for best practices for ensuring high-quality video visits in pediatric subspecialty care. We found novel uses for telemedicine including guided exams and procedures, transparency of patient clinical metrics (labs, growth charts), and areas to explore with patient confidentiality (adolescent Home, Education, Activities, Drugs, Suicidality, and Sex exam). Some of these adaptations are a construct of the technology itself, while others may be due to the patient being present in the home environment, and are adaptations that could potentially be incorporated into provider education around standard practices for telemedicine use.

This study begins to explore the advances and challenges of telemedicine by pediatric specialty. It is clear that further research is important to compare video visits with in-person visits, including effectiveness in urgent, new patient, consultative visit types, effects on shared decision-making, use of adjunctive technologies for examination (e.g., auscultation), impact on health disparities (positive or negative), treatment adherence, and clinical and quality of life outcomes. Additional areas of study to consider include patient versus parent-led communication in video visits, which was observed more in the pediatric rheumatology and pediatric orthopedic surgery video visits. This is of particular importance considering that many of these visits dealt with chronic pediatric conditions, and increased patient activation and engagement are associated with improved health outcomes.^35,36^ Although previous studies have begun to explore the impact of screen versus face-to-face provider-to-patient communication,^37^ future work should explore this in pediatric care.

Limitations for this study included a relatively small sample size (both provider and patient) over a relatively short period of time to make statistically significant statements. Sampling bias also may be a limitation; the subjects participating in this study were able to successfully complete their scheduled telemedicine visit. Further large multicenter studies are needed to be able to generalize these results. This study was not designed to evaluate the reliability of specific exam components in comparison to in-person clinical visits. In addition, this study was not designed to address one of the most important barriers yet potential opportunities for telemedicine, which is improving access for vulnerable populations and decreasing health disparities due to race/ethnicity, socioeconomic status, rural communities, and disabilities. This study represents the first of its kind with understanding telemedicine use from multiple perspectives in pediatric subspecialty medicine—and although these video visits were not done due to patient-specific COVID-19-related health concerns—we have begun to be able to understand telemedicine application in high-volume, routine pediatric subspecialty care.

## Conclusion

This study describes telemedicine use in pediatric subspecialties with respect to patient–provider communication, patient satisfaction, provider experience, and clinical features during the COVID-19 pandemic. It is the first study of its kind to simultaneously evaluate telemedicine performance in four different pediatric specialties and demonstrates a use case for telemedicine in follow-up pediatric subspecialty care. Although telemedicine use has skyrocketed with the pandemic, demonstrating feasibility, additional research is needed to address its acceptability and safety as part of routine pediatric subspecialty care. The hope is to be able to include telemedicine as a permanent piece in the health care delivery puzzle. More in-depth studies are needed to better understand patient and provider engagement with telemedicine, specifically, how it affects patient-reported health outcomes, patient activation, and shared decision-making in pediatric chronic care. Likewise, further research needs to be done to address novel approaches using telemedicine that may include technical interventions and provider education models. In doing so, we may begin to advance telemedicine so that we can standardize its practices, optimize provider and patient education, and improve clinical outcomes—COVID or not.

## Supplementary information

Supplementary Materials
