# Supplementary material for: Pediatric subspecialty telemedicine use from the patient and provider perspective
Source: Pediatr Res. 2021 Mar 22;91(1):241–6. doi: 10.1038/s41390-021-01443-4 (PMC7984505; doi:10.1038/s41390-021-01443-4)
Supplement: Supplementary file 1 — Supplementary Materials [file 41390_2021_1443_MOESM1_ESM.pdf]

SUPPLEMENTARY MATERIALS

Figure 1. Observation Checklist

Who gave the history/answered the questions >50% : Patient versus Parent

OBSERVATION	YES	NO
Issue with video visit logistics (audio/video/both)		
Check-in process?		
Focused physical exam?		
If yes, which components?		
Vitals provided by patient?		
If yes, which components?		
Labs delayed? (Labs to be done following visit)		
Imaging delayed? (needs to be done following visit)		
Was this a multi-disciplinary visit?		
Need for social work		
Medical plan stated by provider?		
Provider shared labs/imaging results		
Anyone else join the visit?		
If yes, who?		
Novel actions done in visit? (other observations)		
If yes, please describe		

Follow up includes future telehealth?		

Figure 2. Provider Survey

Provider Telemedicine Survey

Start of Block: Background Info

Q1 Provider Name

____

Q3 Encounter Date

____

Q4 Department

____

Q5 Were you able to elicit all the pertinent information needed to make clinical assessments for today's video visits?

- ◯ Yes
- ◯ No

Display This Question:

If Were you able to elicit all the pertinent information needed to make clinical assessments for tod... = No

Q6 1) If NO to above, what additional information was needed?

____

____

____

Q7 Were the patients able to conduct the visit in a quiet/visible setting?

◯ Yes

◯ No

Display This Question:

If Were the patients able to conduct the visit in a quiet/visible setting? = No

Q8 1) If NO to above, what was the issue?

Q9 Were any components of the physical exam reliably done by video?

◯ Yes

◯ No

Display This Question:

If Were any components of the physical exam reliably done by video? = Yes

Q10 If YES to above, which components of the exam? (ex: vitals, joint exam, eye exam)

Q11 Were the visits done today converted to telehealth given recent COVID-19 outbreak?

◯ Yes

◯ No

◯ Unknown

Q12 In the setting of recent public health concerns, do you think it was it safer for your patients to conduct these visits by video rather than in person?

◯ Yes

◯ No

Q15 How would you rate the overall quality of this video visits done today?

	Poor	Excellent
Click to write Choice 1		

Q13 Please provide any additional comments regarding your video visit experience:

____

____

____

____

____

End of Block: Background Info
